# Symptom and Age Homophilies in SARS-CoV-2 Transmission Networks during the Early Phase of the Pandemic in Japan

**DOI:** 10.3390/biology10060499

**Published:** 2021-06-03

**Authors:** Ali Andalibi, Naoru Koizumi, Meng-Hao Li, Abu Bakkar Siddique

**Affiliations:** 1 College of Science, George Mason University, Fairfax, VA 22030, USA; aandalib@gmu.edu; 2 Schar School of Policy and Government, George Mason University, Arlington, VA 22201, USA; mli11@gmu.edu (M.-H.L.); asiddi@gmu.edu (A.B.S.)

**Keywords:** SAR-COV-2, COVID-19, asymptomatic patients, viral transmission networks, exponential random graph model (ERGM) network analysis, demographic homogeneities and heterogeneities, symptomological homogeneities and heterogeneities

## Abstract

**Simple Summary:**

In the early stages of the COVID-19 pandemic, Japan conducted contact tracing extensively and published detailed records of thousands of anonymized patients. We leveraged the registry data to perform an exponential random graph model (ERGM) network analysis to examine demographic and symptomological homophilies of SARS-CoV-2 transmission networks in Hokkaido and Kanagawa. Our analysis showed: (1) Age, symptom, and asymptomatic status homophilies in both prefectures; (2) Asymptomatic infections increased as the virus was passed from primary cases to secondary and tertiary ones; (3) Transmission was mostly seen at the primary and secondary levels, with none occurring beyond quaternary; (4) Transmission occurred primarily in healthcare settings, as well as in families.

**Abstract:**

Kanagawa and Hokkaido were affected by COVID-19 in the early stage of the pandemic. Japan’s initial response included contact tracing and PCR analysis on anyone who was suspected of having been exposed to SARS-CoV-2. In this retrospective study, we analyzed publicly available COVID-19 registry data from Kanagawa and Hokkaido (*n* = 4392). Exponential random graph model (ERGM) network analysis was performed to examine demographic and symptomological homophilies. Age, symptomatic, and asymptomatic status homophilies were seen in both prefectures. Symptom homophilies suggest that nuanced genetic differences in the virus may affect its epithelial cell type range and can result in the diversity of symptoms seen in individuals infected by SARS-CoV-2. Environmental variables such as temperature and humidity may also play a role in the overall pathogenesis of the virus. A higher level of asymptomatic transmission was observed in Kanagawa. Moreover, patients who contracted the virus through secondary or tertiary contacts were shown to be asymptomatic more frequently than those who contracted it from primary cases. Additionally, most of the transmissions stopped at the primary and secondary levels. As expected, significant viral transmission was seen in healthcare settings.

## 1. Introduction

Epidemiological studies of COVID-19 have provided mounting evidence that a significant number of individuals infected with SARS-CoV-2 are asymptomatic [1,2] while demonstrating that the symptomology of the disease largely depends on age, sex, and comorbidities [3,4,5]. However, there is limited information on the characteristics of viral transmission networks, especially concerning the demographic and symptomological homogeneities and heterogeneities in viral transmission [6]. To examine the characteristics of SARS-CoV-2 viral transmission, we analyzed Japanese contact tracing data that recorded viral transmission chains as well as demographic and symptomological information of the PCR-confirmed cases during the early phase of the pandemic.

Since the index case was confirmed on 16 January 2020, the Japanese government has been publishing demographic, clinical, and epidemiological data on each individual who has tested positive for the virus. One unique feature of the data is the transmission paths revealed through the contact tracing efforts of the public health centers (PHCs) [7,8,9]. Although contact tracing had become unfeasible in many parts of Japan after the resurgence of the disease in summer 2020, such data were fairly complete and reliable for the first 6 months of the pandemic, i.e., February through July. Under this government-led contact tracing effort known as “cluster countermeasure”, the PHCs retrospectively queried all identifiable individuals who had had in-person contact with a confirmed case during the prior 14 days [8,10,11]. Those who were determined to have been in “close contact” were all subjected to a PCR screening test irrespective of the presence of COVID-19 related symptoms [11]. The criteria used to determine “close contact” included: (i) being a cohabitant of the confirmed case; (ii) having spent long hours in an indoor setting (including a car or an airplane) with the confirmed case; (iii) having provided (medical, nursing) care to the confirmed case without adequate personal protective equipment; (iv) likely exposure to droplets or other body fluids of the confirmed case; (v) having been within 1 m (6 feet) radius of the confirmed case for a total of 15 min or more without protection. Those who did not meet any of these criteria were requested to self-quarantine for 14 days and were advised to receive a test if any symptoms appeared during the 14 days [8,10,11].

We utilized publicly available data from the two prefectures, Hokkaido [12] and Kanagawa [13], for the period when the data was most complete, i.e., between mid-February and mid-July for Hokkaido and between mid-January and early August for Kanagawa. We selected Hokkaido as it was one of the first prefectures that experienced the COVID-19 pandemic and which issued the Declaration of a New Coronavirus Emergency as early as 28 February 2020 [14]. Kanagawa is another prefecture that experienced the pandemic early, with a resident returning from Wuhan, China, and became the country’s index/first case of COVID-19 [15].

The primary objective of the current study was to construct SARS-CoV-2 transmission networks and to analyze the characteristics of viral transmission both descriptively and statistically. In particular, we examined symptom, age, and sex “homophilies”, i.e., whether an infector (the source patient) and the infectee(s) tended to experience similar symptoms, be both asymptomatic or belong to the same age or sex group. Although the results of such analyses do not provide direct evidence about the variations of the virus, the findings shed light on the heterogeneity of SARS-CoV-2 transmission that may be partly explained by viral variants, as well as by how government intervention strategies and the population’s behavior at the time of the pandemic influenced the spread of the virus.

## 2. Materials and Methods

### 2.1. Data

We queried the government registry data for Hokkaido and Kanagawa prefectures. The registry data from Hokkaido contained 1269 cases (including 674 or 53% females and 595 or 47% males) covering the period between 14 February and 22 July 2020, while the data from Kanagawa contained 3123 (including 1346 or 43% females and 1777 or 57% males) cases covering the period between 15 January and 6 August 2020. The final data contained information about 4392 (2020 or 46% female and 2372 or 54%) patients. These cases were originally confirmed by the local PHCs that report to the Ministry of Health, Labor, and Welfare. The Ministry standardizes and publishes the data it receives from the PHCs as part of the comprehensive data contained in the national registry [7]. Individual prefectures also publish the data through their websites, although the specifics and the format of such information vary by prefecture. The data collected by the PHCs during the study period included basic demographic, symptomatological, and epidemiologic information, including the transmission paths (likely infectors and infectees) and travel history of the confirmed cases, with informed consents [7]. We queried both national and local registry data for this study.

The final data included the information on sex; age (<10, 11–20, 21–30, 31–40, 41–50, 51–60, 61–70, 71–80, 81–90, 91–100, or >100); the city of residence, or the testing site; the dates of PCR and the onset of symptoms; symptoms experienced (if any). In Kanagawa, 100 patients were non-Japanese citizens who resided on a US military base. Symptomatological data on these patients were not publicly available, thereby reducing the sample size to 3023 for the analysis of symptomatological data. Similarly, 48 patients did not provide age, reducing the sample size for the analysis involving age to 4344. Data on viral transmission paths were available for 1365 patients (371 cases (29%) and 994 (32%) cases in Hokkaido and Kanagawa, respectively). After excluding those patients whose symptomatological data were missing, 1310 patients (355 patients in Hokkaido and 955 in Kanagawa) remained in the viral transmission networks. For Kanagawa, the likely settings through which transmission occurred were also available for 457 (15%) patients. These included: (i) at medical facilities; (ii) through family; (iii) through friends; (iv) at work; (v) through travel (domestic or international, where the destinations of international travels included: Middle East, South Asia, EU, USA, and others).

### 2.2. Methods

Patient characteristics observed in Hokkaido and Kanagawa were compared using t-tests for continuous and chi-square tests for nominal variables. Depending on the distribution of a continuous variable and the sample size of a nominal variable, Wilcoxon Mann–Whitney and Fisher’s exact tests were used to replace *t-* and chi-square tests, respectively. To investigate the factors correlated with viral transmission and asymptomatic states, logistic regression was performed with the binary dependent variables recording the presence of either viral transmission or asymptomatic states. The factors explaining the viral transmission counts were examined using Poisson regression with the number of infectees per patient as the dependent variable. To examine the difference between the two prefectures, the interaction term between Kanagawa and asymptomatic status was included in the regression model. For the age analysis, the patients aged between 50 and 59 were the reference group, as the preliminary analysis indicated that the group had the lowest proportion of asymptomatic patients. For the month fixed effects, July was the reference month, as the month signifies the end of the first phase of COVID-19 for both prefectures and the beginning of a second wave for Kanagawa. All statistical analyses were performed in STATA (StataCorp, v14). Statistical significance was defined by *p* ≤ 0.05 unless noted otherwise.

We defined asymptomatic cases as those cases who met at least one of the following criteria: (i) the note in the registry indicated the case as an “asymptomatic patient”; (ii) the note indicated “no symptoms”; (iii) there were no symptoms recorded while all other information (age, sex, dates of PCR, etc.) on the patient were present. While these cases may be pre-symptomatic, the notes in the registry data frequently included updated information, indicating, for instance, “the patient reported a fever of (degree) on (date)” after the initial recording. These updates appeared to have been made during the aforementioned 14 day-monitoring periods. Our definition conforms to the current WHO’s guidelines for the determination of asymptomatic cases, i.e., PCR-positive COVID-19 patients without overt symptoms at the time of the laboratory-confirmed infection.

To visually inspect the patterns of viral transmission, we constructed viral transmission networks using the records of the patients whose infectors or infectees were known in the registry data. The network construction and visualization were done using Gephi (v0.9.2). To examine the prevalence of “homophilies” in the viral transmission networks, we applied exponential random graph models (ERGMs), which are well-established models to statistically analyze social and other network data. We specifically investigated several types of homophilies in the networks including: (i) sex homophily, which represents the situations where an infector and the infectee(s) belonged to the same sex; (ii) age homophily representing the situations where an infector and the infectee(s) belonged to the same age group; (iii) symptom homophily where an infector and the infectee(s) had the same symptom; (iv) asymptomatic homophily where both an infector and the infectee(s) were asymptomatic. The first two analyses were to investigate the demographic homogeneities/heterogeneities in the networks, while the last two were to examine the symptomological homogeneities/heterogeneities.

ERGMs essentially test whether infector-infectee chains with a specific type of homophily were more prevalent than those chains without the homophily, i.e., “heterogeneous” chains, in the networks. The heterogeneous class was the reference group in the analysis. In the homophily analysis of sex, we compared the 2 homophily classes of infector-infectee chains to 1 heterogeneous class. The 2 homophily classes were: (a) the (1,1) class, which represented the chains with the sex homophily, while the heterogeneous class contained both (0,1) and (1,0) cases, representing the chains without sex concordance between infectors and the infectees (Table 1). Asymptomatic homophily was structured and analyzed analogously. In the analysis of age, we combined the age categories into 3 age groups (<30, 30–59, and 60+), and compared 3 homophily chains: a) the (1,1) class representing the transmission between patients aged <30 and aged <30; b) the (2,2) class representing the transmission between patients aged 30–59 and 30–59; c) the (3,3) class representing the transmission between patients aged ≥60 and aged ≥60; d) the heterogeneous class comprised of the (1, 2), (2, 1), (1, 3), (3, 1), (2, 3), and (3, 2) chains. In the homophily analysis of symptoms, 2 classes of homophily chains: (a) the (1,1) class representing the presence of the same symptom between infector and the infectee(s); (b) the lack thereof, i.e., the (0,0) class; were compared to c) the heterogeneous class, which represents both (0,1) and (1,0) chains where either infector or the infectee(s) had the symptom. We combined 15 symptoms to make 4 distinct clinical symptom groups to ensure that each class has a sufficient sample size to detect any statistically meaningful variations across the classes:

- Gastrointestinal issues: Stomach ache, nausea, digestive, diarrhea, vomiting, and loss of appetite;
- Mild/upper respiratory issues: cough, pharyngitis, and rhinitis;
- Severe/lower respiratory issues: pneumonia, phlegm, dyspnea, and fatigue;
- Sensory disruption: loss of taste (ageusia) and loss of smell (anosmia).
- Other symptoms that were not grouped included fever, headache, and body ache. All ERGM analyses were run using the programming language R (R Core Team).

## 3. Results

The result section is structured with the following sub-sections: (1) the comparison between Hokkaido and Kanagawa patient profiles; (2) factors correlated with being asymptomatic; (3) factors correlated with the viral transmission; (4) viral transmission networks; (5) demographic and symptomological homophilies in viral transmission networks.

### 3.1. Hokkaido and Kanagawa Case Comparisons

**Figure 1:**
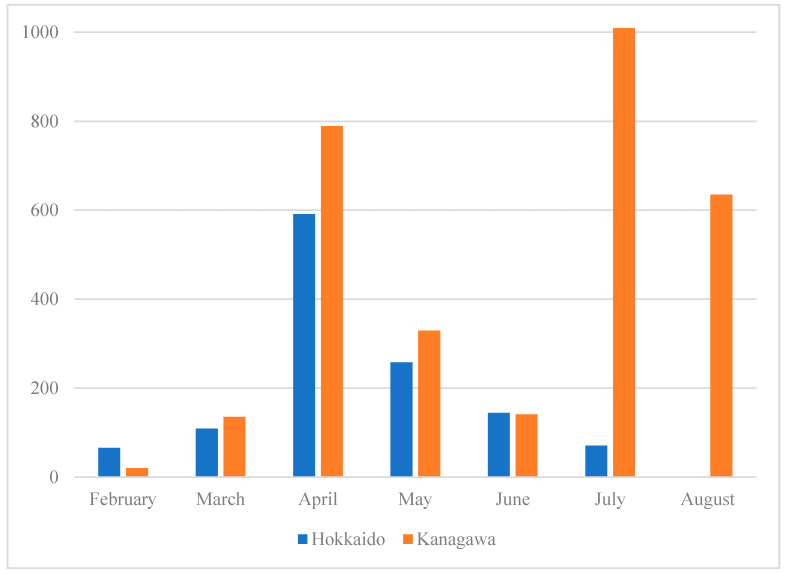
Number of Confirmed COVID-19 Cases by prefectures. The number of COVID-19 cases in Hokkaido and Kanagawa from February 2020 to August 2020. Hokkaido numbers are in blue, and Kanagawa numbers are in orange. Hokkaido’s COVID-19 case numbers peaked in April, corresponding to the first peak of the disease in Kanagawa. There was a second, higher peak in the number of COVID-19 cases in Kanagawa in July.

Figure 1 depicts the number of confirmed cases in each prefecture during the study period. The figure shows that the study period covers the early phases of the pandemic, one between February and June and another after July. A second wave predominantly hit Tokyo and the vicinity, which is part of Kanagawa. Table 2 summarizes the demographic and symptomatological profiles of the patients in the two prefectures.

Overall, the Kanagawa patients were younger (mean age: 39 vs. 54, *p* < 0.001) and were more likely to be asymptomatic (24% vs. 20%, *p* = 0.01) compared to the Hokkaido cases. Among all symptoms experienced in both prefectures, loss of smell (anosmia) and chills were the only symptoms seen more frequently in Kanagawa (14% vs. 2%, *p* < 0.001, for anosmia and 2% vs. 1%, *p* < 0.001, for chills). The symptoms seen more frequently in Hokkaido included rhinitis (16% vs. 8%, *p* < 0.001), fatigue (42% vs. 35%, *p* < 0.001), diarrhea (9% vs. 6%, *p* < 0.001), pneumonia (13% vs. 7%, *p* < 0.001), dyspnea (13% vs. 7%, *p* < 0.001), and body aches (10% vs. 14%, *p* = 0.002). The average number of symptoms experienced (2.96 vs. 2.75, *p* < 0.001) and the average number of infectees per patient (3.65 vs. 1.76, *p* < 0.001) were statistically significantly higher for the Hokkaido patients. The proportion of the patients who infected at least one person was also higher in Hokkaido (17% vs. 13%, *p* = 0.001).

### 3.2. Factors Correlated with Asymptomatic Status

**Figure 2:**
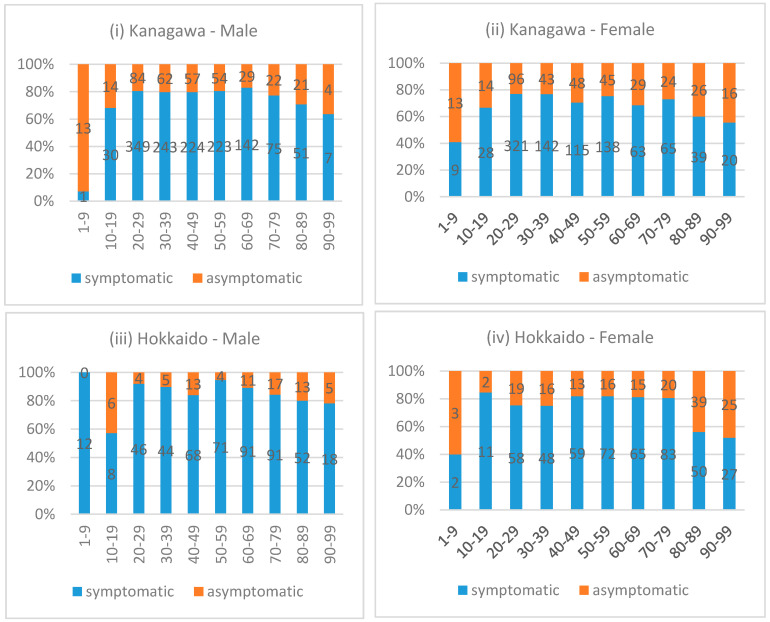
Proportion and the Number of Symptomatic and Asymptomatic Patients by Prefecture and Sex. The proportion and number of symptomatic and asymptomatic COVID-19 patients grouped by age and sex are given for each prefecture. The number of patients showing symptoms is in blue, while asymptomatic numbers are in orange.

Figure 2i–iv presents the proportions of asymptomatic and symptomatic patients by sex and age group in each prefecture. The figures indicate that, for both prefectures, the proportion of asymptomatic patients was higher in both younger (<20) and older (≥70 or 80 depending on the prefecture/sex) generations compared to the middle-aged group, irrespective of sex.

We statistically examined the relationship between age and the likelihood of being asymptomatic, adjusting for patient’s sex using a logistic regression on the data from both prefectures (Table 3). The results were consistent with the observations from Figure 2, demonstrating that, compared to the patients aged between 50 and 59 (the reference age group), the patients aged between 1 and 9 and between 10 and 19 were 4.65 and 1.84 times more likely to be asymptomatic, respectively (*p* < 0.001 for both age groups). Similarly, the patients aged between 80 and 89 as well as 90 and above were 2.18 (*p* < 0.001) and 2.62 (*p* < 0.001) times more likely to be asymptomatic, respectively, compared to the reference group (i.e., 50–59). Males were about 34% less likely to be asymptomatic compared to females (OR = 0.66, *p* < 0.001), and Kanagawa patients were 41% more likely to be asymptomatic (OR = 1.41, *p* < 0.001). With respect to the seasonal effects, using the number of COVID-19 cases in July as the comparator, we found that the likelihood of observing asymptomatic patients was lower in March (OR = 0.23, *p* < 0.001) and April (OR = 0.97, *p* = 0.02), but was higher in May (OR = 1.42, *p* = 0.01) and June (OR = 2.02, *p* < 0.001). The likelihood was also higher in August (OR = 1.29, *p* = 0.04), although the observations for August were from Kanagawa only. There were no asymptomatic cases reported in February (*n* = 86).

**Figure 3:**
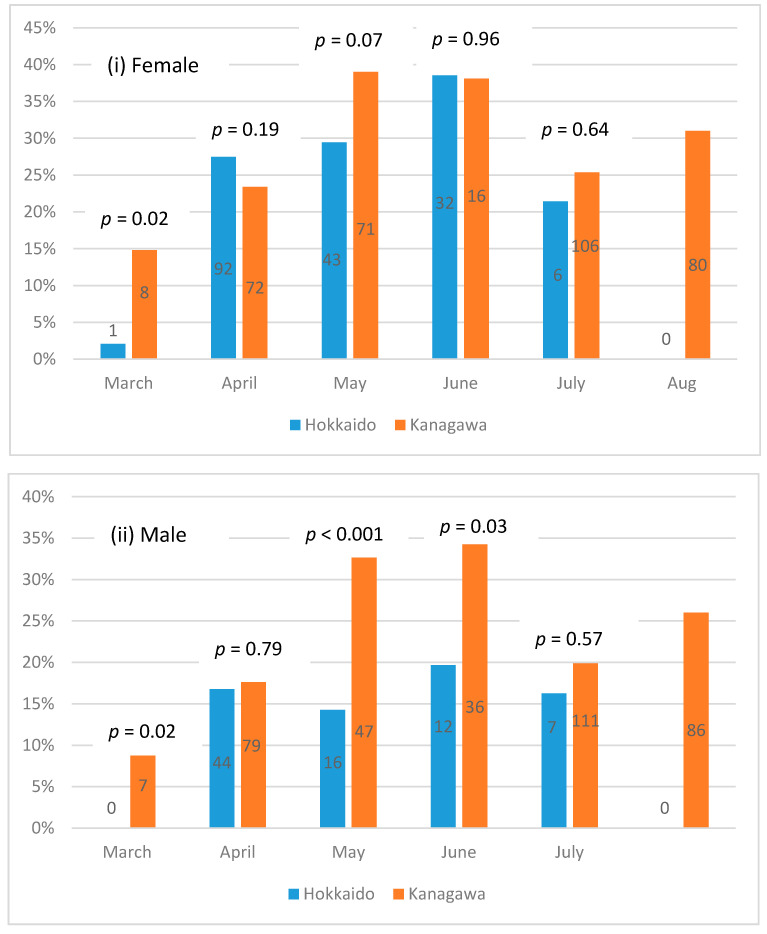
Proportion and Number of Asymptomatic Patients by Prefecture, Month, and Sex. The number of asymptomatic patients of each sex, in Hokkaido and Kanagawa, is shown for each month.

To better understand the seasonal effect, we separated the data by prefectures to examine whether the proportion of the asymptomatic cases varied by month in each prefecture, adjusting for sex (Figure 3i,ii). The *p*-values in the figures correspond to the hypothesis testing examining the equal rate of asymptomatic patients between the two prefectures for the month (Figure 3). The *p*-value could not be computed for August as the data contained only Kanagawa observations. The numbers in the bar charts represent the numbers of asymptomatic patients. The proportion of the asymptomatic patients differed between the prefectures for March for both sexes (*p* = 0.02). Additionally, for males, the proportion was statistically significantly higher in Kanagawa for May (*p* < 0.001) and June (*p* = 0.03). For females, the proportion was higher in Kanagawa for May with a 10% significance level (*p* = 0.07). Overall, Kanagawa demonstrated an upward trend of the asymptomatic case proportion between March and June. The proportion dropped in July and increased again in August. The trend was less clear for Hokkaido, although the proportion of asymptomatic female patients showed an upward trend between March and June.

### 3.3. Factors Correlated with Viral Transmission

To identify the factors correlated with the viral transmission, we performed a logistic regression with the binary dependent variable representing the patients who infected at least one individual (Table 4(a), the left panel). The data indicate that, after adjusting for the shown covariates, age did not influence the likelihood of viral transmission, except for the patients who are aged between 20 and 29. These patients were 30% less likely to transmit the virus compared to those aged between 50 and 59 (the reference group, OR = 0.70, *p* = 0.02). The likelihood of viral transmission was statistically significantly lower in May and June compared to July (OR = 0.43 and 0.47, respectively, *p* < 0.001) and again in August (OR = 0.65, *p* = 0.01). The likelihood of viral transmission by asymptomatic patients differed significantly between Hokkaido and Kanagawa. In Hokkaido, asymptomatic patients were 71% more likely to transmit the virus (OR = 1.71, *p* = 0.01) while, in Kanagawa, asymptomatic patients were 85% less likely to transmit the virus compared to their symptomatic counterparts (OR = e ^(ln(1.71) + ln(0.09))^ = 0.15, *p* < 0.001).

The results of the Poisson regression shown in Table 4(b) demonstrate a concordant pattern. In Hokkaido, the average count of infectees per patient was 5.6 times higher among asymptomatic patients compared to symptomatic patients (IRR = 5.61, *p* < 0.001), while in Kanagawa, the average count was about 80% less among asymptomatic patients compared to symptomatic patients (IRR = e ^(ln(5.61)+ln(0.04))^ = 0.20, *p* < 0.001). In both prefectures, the viral transmission rate was higher in April (IRR = 1.79, *p* < 0.01) and lower in May and June (IRR = 0.47 and 0.33, respectively, *p* < 0.001) compared to July. The transmission rate was lower in August than in July (IRR = 0.67, *p* < 0.001). The average count of infectees was 26% higher among males compared to females (IRR = 1.26, *p* < 0.001). In general, younger patients were infecting fewer people (IRR = 0.36, *p* = 0.01 for the age group 1–9; IRR = 0.30, *p* < 0.001 for the age group 10–19; and IRR = 0.78, *p* = 0.03 for the age group 30–39) compared to those aged between 50 and 59 (the reference group), while older patients were infecting more individuals (IRR = 1.55, *p* < 0.001 for the age group 60–69; IRR = 1.77, *p* < 0.001 for the age group 70–79) compared to the patients aged between 50 and 59.

### 3.4. Viral Transmission Networks

Transmission of the virus ranged from one to four levels (primary to quaternary) in both prefectures. Table 5 summarizes the distribution of the viral transmission levels by symptomatic/asymptomatic status. Quaternary transmission was rare, accounting for less than 1% of all cases in the networks in both prefectures. In both prefectures, the incidences of secondary transmission were the highest, accounting for 58% (Hokkaido) to 61% (Kanagawa) of all the cases in the transmission networks.

The distribution differed significantly between symptomatic and asymptomatic patients (*p* = 0.02 for Hokkaido and *p* < 0.001 for Kanagawa). Relative to symptomatic patients, asymptomatic patients were more concentrated in the secondary (71% vs. 54% for Hokkaido, 80% vs. 58% in Kanagawa) and tertiary (12% vs. 11% for Hokkaido, 7% vs. 4% in Kanagawa) transmission, while symptomatic patients were more concentrated in the primary cases (34% vs. 16% in Hokkaido and 38% vs. 13% in Kanagawa). The results of a logistic regression confirmed this (Table 6). Those patients who contracted the virus through the secondary or tertiary transmission were 2.9 (OR = 2.9, *p* < 0.001) and 3.2 (OR = 3.2, *p* < 0.001) times more likely to be asymptomatic than primary cases, respectively.

Transmission networks of the virus are shown. Green circles represent the primary COVID-19 cases, while purple circles represent the secondary cases. Circle sizes denote the impact (number of infectees). Orange and blue circles represent the tertiary and quaternary infectees. Most of the networks consist of only two individuals.

**Figure 4:**
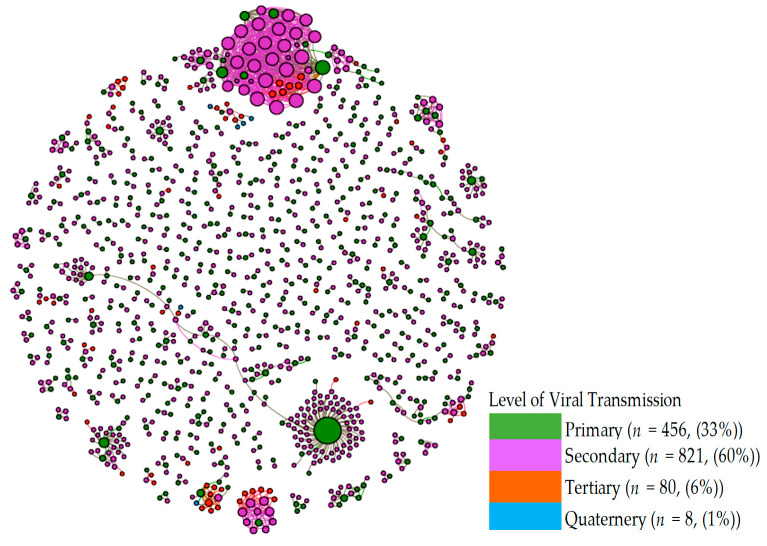
Viral Transmission Level in Viral Transmission Networks (Hokkaido and Kanagawa). Transmission networks of the virus are shown. Green circles represent the primary COVID-19 case, while purple circles represent the secondary cases. Circle size denotes impact (number of infectees). Orange and blue circles represent the tertiary and quaternary infectees. Most of the networks consist of only two individuals.

**Figure A1:**
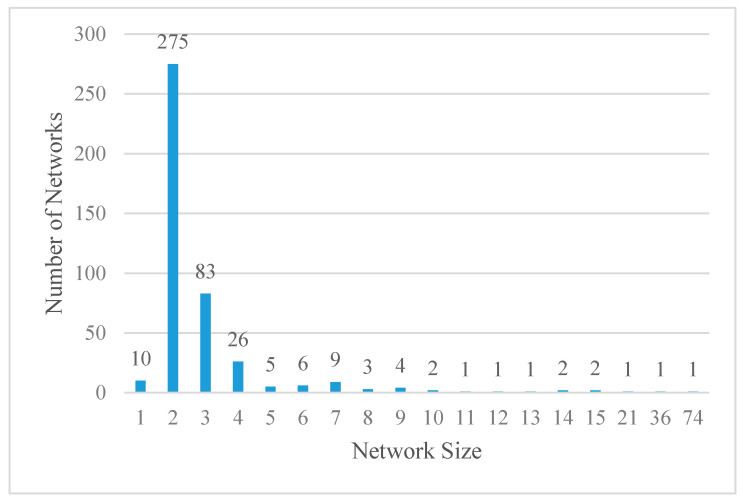
Network size and number.

Figure 4 presents the viral transmission networks by the (color-coded) transmission level for both prefectures. In the diagram, each node represents a patient, while the node size is depicted in proportion to the number of his/her infectees. The transmission networks indicate that the majority of the chains consist of two cases, an infector (green) and an infectee (pink). There are also several large transmission networks in which the virus was spread from a primary infection case (green) to a large number of secondary infection cases (pink). A few networks consisted of several secondary infection cases that spread the virus to tertiary cases (orange). There were a very small number of tertiary infection cases that spread the virus to quaternary cases (blue). Figure A1 in Appendix A shows the histogram of the network sizes presented in Figure 4.

**Figure 5:**
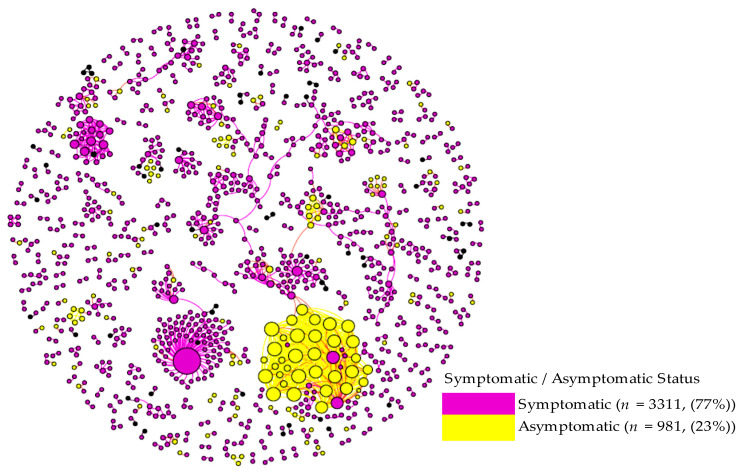
Symptomatic/Asymptomatic Status in Viral Transmission Networks (Hokkaido and Kanagawa). The homophily of asymptomatic and symptomatic cases is shown. Most asymptomatic COVID-19 patients cause downstream infections that are also asymptomatic. Circle size denotes impact (number of infectees).

Figure 5 visualizes the distribution of asymptomatic and symptomatic cases in the viral transmission networks. The figure shows that one cluster (the largest Hokkaido network consisting of 36 cases) was predominantly comprised of asymptomatic cases (33 or 92% asymptomatic and 3 (8%) symptomatic cases). Even excluding this particular cluster, there was a general tendency that asymptomatic cases were more likely to generate asymptomatic cases in subsequent transmission chains. We statistically tested this by examining whether the proportions of symptomatic and asymptomatic patients differed depending on the symptomatic/asymptomatic status of the infectors. The result revealed that approximately 8% of patients infected by symptomatic patients were asymptomatic, while 29% of patients infected by asymptomatic patients were also asymptomatic in the networks (*p* < 0.001).

**Figure 6:**
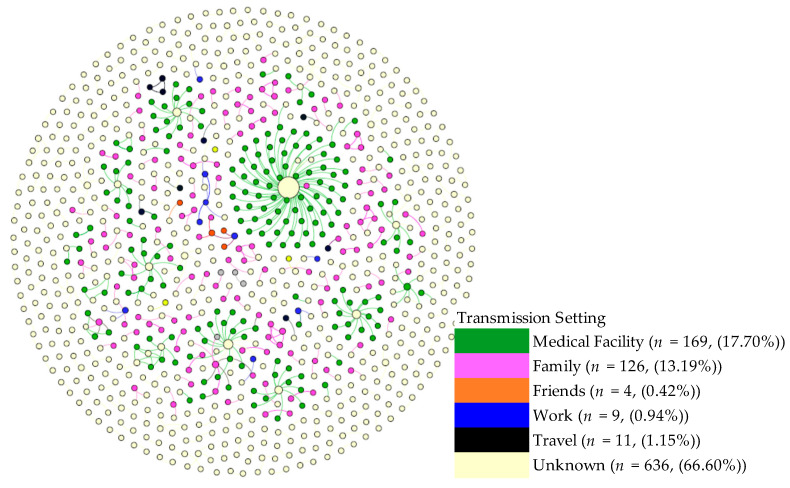
Setting of Viral Transmission in Viral Transmission Networks. The settings in which COVID-19 cases originated are shown. Most frequently, the cases originated in a medical facility (green). Family settings (purple) were also important origination settings for the disease, as were work settings (blue), although to a lesser extent. Circle sizes denote the impact (number of infectees).

Separately, we visualized the transmission networks by age and sex, which revealed no discernable patterns and thus are not presented here. For Kanagawa networks, we also visualized the viral transmission networks by setting (Figure 6). The figure indicates that medical facilities were the dominant setting for viral spread, followed by within family transmissions. The figure clearly shows that the viral transmissions within individual families and in all other settings often generated small chains, each with 1 or 2 subsequent infections, whereas the viral transmissions within each medical facility tended to generate a substantially larger network.

### 3.5. Demographic and Symptomological Homophilies in Viral Transmission Networks

The ERGM analyses examined the prevalence of sex, age, and symptom homophilies in the viral transmission networks. Table A1 in Appendix A provides the number of chains in each of the sex, age, and symptom classes observed in the Kanagawa and Hokkaido viral transmission networks. Table 7 presents the results of the ERGM analysis. As evidenced by the odds ratios of 1 or above, homophily chains were more prevalent than heterogeneous chains in general. The only exception was the gastrointestinal homophily in Kanagawa (OR = 0.36, *p* < 0.001), which indicated that the gastrointestinal homophily chains were 64% less likely than the heterogeneous chains. The gastrointestinal homophily chains were more likely than the heterogeneous chains in Hokkaido (OR = 2.20, *p* < 0.001), showing differences in disease manifestation between the two prefectures. For all other homophilies, the results were consistent between the prefectures. In particular, the asymptomatic homophily and the sensory disruption homophily chains were statistically more likely than the heterogeneous chains in both prefectures. Concerning the asymptomatic homophily, the asymptomatic chains were 5.21 times and 3.67 times more likely than the heterogeneous chains in Hokkaido (OR = 5.21, *p* < 0.001) and Kanagawa (OR = 3.67, *p* < 0.001), respectively. Regarding the sensory disruption homophily, the chains were 2.02 and 2.09 times more likely in Hokkaido (OR = 2.02, *p* = 0.03) and Kanagawa (OR = 2.09, *p* = 0.002), respectively. The fever homophily chains were also more likely in both prefectures (OR = 2.00, *p* < 0.001 for Hokkaido, and OR = 1.49, *p* < 0.001 for Kanagawa), although for Hokkaido, no-fever homophily chains (0,0) was also more likely (OR = 4.13, *p* < 0.001). Several additional (0,0) class homophilies were significant in Hokkaido. These included body ache (OR = 1.94, *p* < 0.001), mild/upper respiratory issues (OR = 2.45, *p* < 0.001), and severe/lower respiratory issues (OR = 2.09, *p* < 0.001). There was no statistically significant sex homophily in either prefecture (p *>* 0.10). In terms of the age homophily, the age ≥60 homophily was observed in both prefectures, indicating that viral transmissions between older (≥60) patients were more likely (OR = 1.40, *p* < 0.001 in Hokkaido, and OR = 3.19, *p* < 0.001 for Kanagawa). In addition, the Kanagawa networks indicated the presence of age <30 (OR = 2.58, *p* < 0.001) and 31–59 (OR = 1.82, *p* < 0.01) homophilies.

## 4. Discussion

The current retrospective study analyzed publicly available secondary data of 4392 (2020 females and 2372 males) individuals who were PCR-positive for SARS-CoV-2. The comparison of the results from the two prefectures has shown similarities, as well as differences. In both prefectures, asymptomatic cases were about 20% and were more likely to be female, and in either the younger (<20) or older (≥80) age group. The rate of asymptomatic infection observed in the current study is comparable to that report in prior literature [16,17,18]. The evidence that female patients are more likely to be asymptomatic is also relatively well-established [19,20], although these studies also indicate that younger female patients are particularly more likely to be asymptomatic. The observation made in the current study that older patients are more likely to be asymptomatic might be unique to Japan. Japan is known as one of the world’s top countries for longevity, especially in females [21]. Such prolonged life expectancy has been accompanied by concomitant improvement in overall health and physical functions in the older population, reducing the mortality rate in Japanese female centenarians even further in the last decade [21,22]. Moreover, studies have shown that the Japanese elderly population, as a whole, is lean, with a low body mass index (BMI), which is associated with longevity [23,24]. Additionally, the susceptibility of overweight individuals, who often suffer from diabetes and hypertension to severe COVID-19 disease, has been established in multiple studies [25]. Our analysis also shows that, regardless of showing symptoms, in both prefectures, males transmitted the virus at a higher rate. This is consistent with the results of other studies that have shown a slower ability to clear viral RNA in males versus females and a more efficient immune response in females [26,27,28].

The primary difference observed between the prefectures was the viral transmission rate among asymptomatic patients. In Hokkaido, asymptomatic patients were more likely to transmit the disease, while, in Kanagawa, symptomatic patients were more likely to transmit the virus. Other studies have also reported varying results about the viral transmissions by symptomatic and asymptomatic cases, ranging between 0% and 2.2% for asymptomatic transmission and between 0.8% to 15.4% for symptomatic transmission [29,30,31,32,33]. The most recent meta-analysis reports that the relative risk of asymptomatic transmission was 42% lower than that of symptomatic transmission [18]. The higher viral transmission by asymptomatic cases in Hokkaido may reflect the fact that, during the early stages of the pandemic, the presence of asymptomatic infections as well as the risk of subsequent transmissions by asymptomatic cases were less known in the population, and thus the maintenance of in-person social contacts by asymptomatic cases was more widespread in Hokkaido than in Kanagawa during the late spring and summer.

Another explanation may be the differences in the climate and temperature. Hokkaido is farther north and significantly colder than Kanagawa, especially during the winter, and experienced its first COVID cases during the winter months, peaking in April (mean temperature 5 °C). Given that the seasonality of respiratory viral diseases and the impact of temperature and humidity on the body’s response to these pathogens is well-established [34], it stands to reason that symptomatic respiratory diseases such as COVID-19 may be more prevalent and associated with more severe symptoms, in the colder clime of Hokkaido than in the warmer temperatures of Kanagawa. As such, Hokkaido patients would have been more easily identified and quarantined, thus resulting in a reduction in the transmission from symptomatic patients relative to asymptomatic ones. In Kanagawa, on the other hand, environmental factors such as the warmer temperatures during the latter two COVID peaks in July and August could have resulted in lower viral shedding from asymptomatic carriers, thus resulting in a lower observed transmission from this group.

Consistent with other studies, our network analysis showed that, both in Hokkaido and Kanagawa, nosocomial infections gave rise to large transmission networks (36 cases in Hokkaido and 74 cases in Kanagawa). High levels of SARS-CoV-2 transmission in health care settings have been observed by others as well [35,36,37,38], especially in the early stage of the pandemic when proper protection of health care workers was not in place. The role of super-spreaders in the indoor setting has been well documented [39,40,41]. Several explanations have been provided regarding the existence of super-spreaders, including: (i) high viral shedding of the seed case due to low immunocompetence, attributable to underlying medical conditions or co-infection; (ii) the indoor environmental factors, such as humidity, which are conducive to epithelial innate immune function, resulting in higher levels of viral replication and shedding; (iii) active social behavior of the seed case [42,43,44,45,46].

Transmission clustering has also been reported in the family setting. These studies have shown that within-family transmissions are often localized and that the risk of transmission in the setting is comparatively high [6]. Our study also found clustering within families, although the clusters were small. Moreover, with the exceptions of the two medical facility transmission networks, our analysis revealed that the majority (64%) of the networks were comprised of two patients (an infector and an infectee), and more than 90% of the networks involved less than five patients. In recent months, more evidence on the makeup of SARS-CoV-2 transmission lineages has become available [47,48,49]. These studies report that the proportion of the lineages that go beyond secondary transmissions is surprisingly low, in part driven by lockdowns and the implementation of effective interventions to control the pandemic. For instance, consistent with our data, Geoghegan et al. (2020) report that less than 20% of virus introductions into New Zealand generated viral transmission of more than one additional case. Here, it is possible that a geographic attribute (being an island) of the two countries may have resulted in similar intervention effects.

To our knowledge, no prior studies have examined demographic and symptomological homophilies of the SARS-CoV-2 viral transmission networks. Homophilies, in this case, refers to the similarities between the infector and infectee. Our ERGM analysis revealed the presence of age homophily among older (≥60) patients in both prefectures. This may be at least partially attributable to the age grouping of individuals in nursing homes and care facilities, as well as the forms of social interactions (e.g., indoor rather than outdoor, duration, etc.) among older adults, which may have led to more viral transmission to their confreres. In Kanagawa, additional homophilies were detected in the patients’ aged <30 and 31–59, likely reflecting the generational differences in social behavior, especially in an urban setting such as Kanagawa.

In addition to age homophily, we also observed symptomatic and asymptomatic homophilies. Symptomatic infectors were more likely to give rise to symptomatic infectees, while patients who got the disease from an asymptomatic infector were likely to also be asymptomatic. Although the reason behind this homophily remains unclear, it could be the result of a lower viral load in patients with mild disease, which would result in fewer shed viral particles and a consequent lower infectious dose delivered to an infectee. However, whether asymptomatic patients have a lower viral load is controversial, with some studies showing lower levels and others showing no difference [50,51]. Related to this point, we also observed that those patients who contracted the virus through secondary or tertiary transmission were more likely to be asymptomatic than primary cases, potentially suggesting natural viral attenuation. Unfortunately, no sequence data were available for the cases used in our study, and therefore it was impossible to provide more definitive reasons for the observed homophilies. Future epidemiological studies could benefit from the sequencing of viral isolates from primary and higher-level cases to determine whether symptom homophilies exist within individual lineages.

Homophily of sensory disruption (i.e., anosmia and ageusia) was observed in the networks of both prefectures. Moreover, we observed that homophily chains were more prevalent than heterogeneous chains in the network. These findings suggest that genetic variations of SARS-CoV-2 may be underlying the variance in symptoms and that the transmission of virions from a particular genetic lineage from an infector to an infectee may result in a similarity of symptoms between these two groups. Phylogenetic analyses of SARS-CoV-2 sequences from these cases are warranted to explore this hypothesis.

The study has several limitations in addition to the aforementioned unavailability of viral samples. First, the current study is a retrospective secondary data analysis, and thus, the authors are unable to ensure the quality of the data. In particular, the viral transmission data are subject to systematic bias if contact tracing was performed disproportionately in specific cases or cohorts. The guideline published by the Japanese government stipulates that all individuals who were in “close contact” with the confirmed cases be subject to an “initial (PCR) screening test”. While it is likely that the guideline was still closely followed during the study period of February to July 2020, it is possible that the level of compliance was somewhat compromised as the pandemic got worsened. It is also possible that individuals in certain settings were followed up more completely than the individuals in other settings due to accessibility. For instance, it is easier to identify those cases who were in “close contact” with the patients in medical facilities than those who were in “close contact” with cases who contracted the virus while traveling. Secondly, as mentioned in the methods section, our asymptomatic patients could include pre-symptomatic cases. Even though the notes in the registry data appeared to have been updated during the 14 day-monitoring periods, we are unable to ensure the completeness of such updates.

## 5. Conclusions

We analyzed the records of 4392 PCR-confirmed COVID-19 patients in two prefectures, Hokkaido and Kanagawa, during the early stages of the pandemic in Japan. The network analysis of the viral transmission chains revealed that demographic and symptomological homophilies exist in both prefectures. In particular, age homophily existed in both prefectures, especially between older adults, but more prevalently in the Tokyo area. No sex homophily was observed in either prefecture. Most importantly, similar patterns of symptom homophilies were seen in both prefectures, with the most striking being the homophily between asymptomatic infectors and infectees. This result substantiates the logic behind contact tracing and testing of “close contact” cases, even in the absence of the symptoms, to contain the spread of the virus. Furthermore, as with COVID-19, control of future pandemics will likely also greatly benefit from public education to promote testing in “close contact” cases, as well as from the establishment of an efficient testing system during the early stages of outbreaks.

## Figures and Tables

**Table 1:** Homophily and Heterogeneous Chains and Classes.

Homophily */Heterogeneous ** Chain Category	Homophily/Heterogeneous Class	Description
Sex	(1,1)	Same sex in infector and infectee
	(1,0), (0,1)	Different sex in infector and infectee
Asymptomatic status	(1,1)	Asymptomatic status concordance
	(1,0), (0,1)	No asymptomatic status concordance
Age (3 age groups: <30, 30–59, and 60+)	(1,1)	Transmission between age <30 and age <30 patients
	(2,2)	Transmission between 30–59 and 30–59 patients
	(3,3)	transmission between ≥60 and age ≥60 patients
	(1,2), (2,1), (1,3), (3,1), (2,3) (3,2)	Transmission between different age groups
Symptoms	(1,1)	Same symptoms in infector and infectee
	(1,0), (0,1)	Different symptoms in infector and infectee

* Homophily chain refers to the situation where an infector and his/her infectee shared the same characteristics. ** Heterogeneous chain refers to the situation where an infector and his/her infectee had different characteristics. Types of homophily in the COVID patient population are shown. Homophily existed when the infector and infectee shared a status. Sex homophily (i.e., infector and infectee are the same sex) is represented by (1,1). The absence of sex homophily (i.e., infector and infectee were of different sexes) is represented by 1,0 and 0,1. Asymptomatic status homophily (i.e., an individual infected by an asymptomatic infector, was also asymptomatic) is represented by 1,1. Absence of asymptomatic status homophily (i.e., either the infector or the infectee had symptoms of COVID). Age homophily (i.e., infector and infectee are the same age group) is represented by (1,1 (infector and infectee are <30 years old); 2,2 (infector and infectee were 30–59 years old)), 3,3 (infector and infectee were >60 years old)). Absence of age homophily (i.e., infector and infectee were in different age groups) is represented by (1,2, 2,1, 1,3, 3,1, 2,3, and 3,2). Symptom homophily (i.e., infector and infectee shared the same symptoms) is represented by (1,1). The absence of symptoms homophily (i.e., infector and infectee had different symptoms) is represented by 1,0 and 0,1.

**Table 2:** COVID-19 Patient Characteristics by Prefecture.

Patient Characteristics	Hokkaido (*n* = 1269)	Kanagawa (*n* = 3023)	*p*-Value
Age, *n* (%)			
1–9	17 (1%)	65 (2%)	0.13
10–19	27 (2%)	89 (3%)	0.23
20–29	127 (10%)	873 (28%)	<0.001
30–39	113 (9%)	498 (16%)	<0.001
40–49	153 (12%)	457 (15%)	0.06
50–59	163 (13%)	473 (15%)	0.11
60–69	182 (15%)	268 (9%)	<0.001
70–79	211 (17%)	202 (6%)	<0.001
80–89	154 (13%)	144 (5%)	<0.001
90–99	75 (6%)	48 (2%)	<0.001
100+	5 (0.4%)	0 (0%)	<0.001
Male, *n* (%)	595 (46.89%)	1777 (56.90%)	<0.001
Symptom ^1^, *n* (%)			
Fever	818 (84%)	1919 (83%)	0.01
Cough	395 (41%)	861 (37%)	0.08
Pharyngitis	174 (18%)	419 (18%)	0.86
Rhinitis	157 (16%)	176 (8%)	<0.001
Fatigue	408 (42%)	814 (35%)	<0.001
Diarrhea	93 (9%)	132 (6%)	<0.001
Headache	185 (19%)	459 (20%)	0.57
Pneumonia	125 (13%)	169 (7%)	<0.001
Dyspnea	123 (13%)	171 (7%)	<0.001
Loss of Taste (ageusia)	167 (17%)	406 (18%)	0.77
Loss of Smell (anosmia)	17 (2%)	320 (14%)	<0.001
Loss of Appetite	18 (2%)	32 (1%)	0.32
Body Aches	131 (14%)	225 (10%)	0.002
Nausea/Vomiting	27 (3%)	43 (2%)	0.10
Phlegm	21 (2%)	71 (3%)	0.15
Chill	5 (1%)	56 (2%)	<0.001
Asymptomatic cases, *n* (%)	257 (20%)	724 (24%)	0.01
Number of symptoms ^1^, mean (SD)	2.96 (1.57)	2.75 (1.33)	<0.001
Number of transmissions, mean (SD)	3.65 (5.41)	1.76 (3.87)	<0.001
Share of those transmitted, *n* (%)	210 (17%)	396 (13%)	0.001

^1^ Excluding asymptomatic cases. The percentage of COVID-19 patients who fell into various groups based on age, type of symptom, number of symptoms, absence of symptoms (asymptomatic), and the number of people infected by and infector (number of transmissions) is provided. The percentages were based on the number of COVID-19 patients in each prefecture (1269 in Hokkaido and 3123 in Kanagawa).

**Table 3:** Logistic Regression for the Determinants of Asymptomatic Status.

Variables	OR	*p*-Value	95% Conf.	Interval
Male	0.66	<0.001	0.56	0.76
Kanagawa	1.41	<0.001	1.16	1.72
Month ^1^				
March	0.23	<0.001	0.13	0.41
April	0.97	0.78	0.78	1.20
May	1.42	0.01	1.10	1.82
June	2.02	<0.001	1.48	2.75
August	1.29	0.04	1.01	1.63
Age Group ^2^				
1–9	4.65	<0.001	2.73	7.91
10–19	1.84	0.01	1.17	2.90
20–29	0.98	0.87	0.75	1.27
30–39	1.04	0.81	0.78	1.38
40–49	1.21	0.19	0.91	1.61
60–69	1.09	0.61	0.79	1.50
70–79	1.09	0.61	0.78	1.52
80–89	2.18	<0.001	1.57	3.04
90–99	2.62	<0.001	1.70	4.03

^1^ Reference month: July, ^2^ Reference age group: 50–59. The calculated odds ratio (OR), *p*-values, and 95% confidence intervals are provided to assess if sex, the month of infection, and age were significantly correlated with the absence of symptoms (asymptomatic status). The reference month chosen was July, which coincided with the highest number of cases in Hokkaido and the first peak in Kanagawa. The reference age group was 50–59, as it had the largest number of patients in both prefectures.

**Table 4:** Logistic Regression for the Determinants of Viral Transmission.

	(a) Logistic Regression on Viral Transmission				(b) Poisson Regression on the Number of Infectees			
Variable	OR	*p*-Value	95%	CI	IRR	*p*-Value	95%	CI
Male	1.17	0.09	0.97	1.41	1.26	<0.001	1.13	1.41
Asymptomatic	1.71	0.01	1.18	2.48	5.61	<0.001	4.82	6.52
Kanagawa	1.12	0.36	0.88	1.42	1.01	0.90	0.87	1.17
Kanagawa x Asymptomatic	0.09	<0.001	0.05	0.16	0.04	<0.001	0.03	0.05
Month ^1^								
February	1.44	0.19	0.83	2.48	1.05	0.83	0.69	1.58
March	1.27	0.21	0.88	1.84	1.04	0.77	0.79	1.37
April	0.88	0.33	0.69	1.13	1.79	<0.001	1.52	2.10
May	0.43	<0.001	0.30	0.62	0.47	<0.001	0.37	0.61
June	0.47	<0.001	0.30	0.76	0.33	<0.001	0.24	0.47
August	0.65	0.01	0.47	0.89	0.67	<0.001	0.52	0.87
Age Group ^2^								
1–9	0.61	0.32	0.23	1.61	0.36	0.01	0.16	0.81
10–19	0.56	0.11	0.28	1.13	0.30	<0.001	0.16	0.53
20–29	0.70	0.02	0.51	0.95	0.95	0.58	0.78	1.15
30–39	1.01	0.95	0.73	1.40	0.78	0.03	0.62	0.97
40–49	0.95	0.78	0.69	1.32	0.85	0.14	0.69	1.05
60–69	1.22	0.25	0.87	1.71	1.55	<0.001	1.28	1.88
70–79	1.09	0.63	0.76	1.58	1.77	<0.001	1.46	2.14
80–89	1.14	0.53	0.76	1.71	0.99	0.90	0.78	1.24
90–99	1.07	0.82	0.60	1.93	0.94	0.69	0.70	1.27

^1^ Reference month: July, ^2^ Reference age group: 50–59. (a) The calculated odds ratio (OR), *p*-values, and 95% confidence intervals are provided to assess if sex and age were significantly correlated with the ability to transmit the virus (viral transmission). (b) Poisson regression was performed on the number of people infected by a given infector (i.e., the number of infected individuals whose infection could be definitively contact traced back to a particular individual with COVID-19). The calculated incidence rate ratio (IRR), *p*-values, and 95% confidence intervals are provided. The reference month chosen was July, which coincided with the highest number of cases in Hokkaido and the first peak in Kanagawa. The reference age group was 50–59, as it had the largest number of patients in both prefectures.

**Table 5:** Distribution of Viral Transmission Levels by Prefecture and Symptomatic/Asymptomatic Status.

Level	Hokkaido			Kanagawa		
	Symptomatic	Asymptomatic	Total	Symptomatic	Asymptomatic	Total
Primary	96 (34.3%)	12 (16%)	108 (30.4%)	322 (38%)	13 (13%)	335 (35.1%)
Secondary	152 (54.3%)	53 (70.7%)	205 (57.8%)	497 (58%)	82 (80%)	579 (60.6%)
Tertiary	30 (10.7%)	9 (12%)	39 (11.0%)	32 (3.8%)	7 (7%)	39 (4.1%)
Quaternary	2 (0.7%)	1 (1.3%)	3 (0.8%)	2 (0.2%)	0 (0%)	2 (0.2%)
Total	280 (100%)	75 (100%)	355 (100%)	856 (100%)	102 (100%)	955 (100%)
*p*-value *	0.02			<0.001		

* Fisher’s exact test, This table summarizes the level of transmission of the virus based on contact tracing. The original infector is considered to be the primary case. Secondary transmission occurred when the primary COVID-19 case (the primary symptomatic and asymptomatic) infects a naïve individual (the secondary infectee). Tertiary transmission occurred when the secondary infectee passes on the virus to a naïve individual (tertiary infectee). Quaternary transmission occurred when the tertiary infectee passes on the virus to a naïve individual (quaternary infectee).

**Table 6:** Logistic Regression for the Likelihood of Asymptomatic Status by Viral Transmission Level.

Level *	OR	*p*-value	95% Conf.	Interval
Secondary	2.90	<0.001	1.84	4.56
Tertiary	3.24	<0.001	1.61	6.49
Quaternary	2.58	0.41	0.28	23.98

* Reference group: Primary cases, This table summarizes the results of the logistic regression analysis to determine if asymptomatic carriers were more likely to result in secondary, tertiary, or quaternary infectees. The calculated odds ratio (OR), *p*-values, and 95% confidence intervals are provided to assess transmission levels.

**Table 7:** Sex, Age, and Symptom Homophilies in Viral Transmission Networks by Prefecture.

Variable	Homophily Class *	Hokkaido				Kanagawa			
		OR	*p*-Value	95%	CI	OR	*p*-Value	95%	CI
Sex	Female: (0,0)	0.92	0.38	0.77	1.11	0.98	0.83	0.81	1.18
	Male: (1,1)	1.08	0.42	0.90	1.29	0.88	0.18	0.72	1.06
Age	<30: (1,1)	1.19	0.41	0.78	1.82	2.58	<0.001	2.03	3.29
	30–59: (2,2)	1.05	0.74	0.8	1.36	1.82	<0.001	1.5	2.21
	≥60 (3,3)	1.40	<0.001	1.19	1.66	3.19	<0.001	2.55	3.99
Asymptomatic status	No: (0,0)	1.53	0.03	1.04	2.24	0.97	0.87	0.7	1.35
	Yes: (1,1)	5.21	<0.001	3.75	7.24	3.67	<0.001	1.97	6.84
Fever	No: (0,0)	4.13	<0.001	2.95	5.78	1.18	0.33	0.85	1.65
	Yes: (1,1)	2.00	<0.001	1.43	2.79	1.49	<0.001	1.21	1.84
Headache	No: (0,0)	1.18	0.35	0.84	1.65	0.89	0.37	0.7	1.14
	Yes: (1,1)	0.62	0.36	0.22	1.75	1.62	0.06	0.98	2.68
Body ache	No: (0,0)	1.94	<0.001	1.36	2.78	0.86	0.28	0.65	1.13
	Yes: (1,1)	1.82	0.12	0.86	3.88	2.31	0.04	1.05	5.09
Gastrointestinal issues	No: (0,0)	2.20	<0.001	1.50	3.21	0.36	<0.001	0.29	0.46
	Yes: (1,1)	1.34	0.59	0.46	3.85	1.71	0.12	0.86	3.4
Mild/upper respiratory issues	No: (0,0)	2.45	<0.001	1.87	3.21	0.95	0.66	0.76	1.18
	Yes: (1,1)	0.89	0.5	0.63	1.26	1.60	<0.001	1.26	2.02
Severe/lower respiratory issues	No: (0,0)	2.09	<0.001	1.6	2.72	0.82	0.08	0.67	1.02
	Yes: (1,1)	0.95	0.78	0.67	1.34	1.12	0.43	0.85	1.47
Sensory disruption	No: (0,0)	1.64	0.01	1.18	2.26	0.82	0.09	0.65	1.03
	Yes: (1,1)	2.02	0.03	1.09	3.76	2.09	0.002	1.32	3.32

* Homophily chain refers to the situation where an infector and his/her infectee shares the same characteristics. The reference group was a heterogeneous class, i.e., the chains that are not homophily. This table summarizes the results of the logistic regression analysis to determine if homophily comprises a significant aspect of viral transmission networks in Hokkaido and Kanagawa. The calculated odds ratio (OR), *p*-values, and 95% confidence intervals are provided to assess homophily in age, sex, symptoms, and asymptomatic status. The symptom homophily classes assessed were fever, headache, body ache, gastrointestinal issues (nausea and vomiting), upper respiratory involvement (cough, sneezing, and rhinitis), lower respiratory involvement (dyspnea), and sensory disruption (anosmia and ageusia).

## Data Availability

The data presented in this study are openly available from the COVID-19 registry in Hokkaido [http://www.pref.hokkaido.lg.jp/hf/kth/kak/hasseijoukyou.htm, accessed on 19 May 2021] and Kanagawa [https://www.pref.kanagawa.jp/docs/ga4/covid19/occurrence.html, accessed on 19 May 2021], Japan. Cleaned, formatted data used for the statistical analysis are available upon request from the corresponding author.

## Appendix A

Figure A1 shows the histogram of the network size. The figure demonstrates that more than 90% (i.e., (10 + 275 + 83 + 26) / 433 = 91%) of the networks were comprised of 4 patients or less. In particular, more than 60% of the networks were comprised of only 2 patients (275 / 433 = 64%), an infector and an infectee. The two largest networks that contained 74 and 36 cases were attributable to nosocomial infections in Kanagawa and Hokkaido, respectively. The Kanagawa network developed in April (*n* = 28) and May (*n* = 46), 2020, while the Hokkaido network evolved in April 2020.

The distribution of the size of networks is shown. Networks consisting of two individuals make up the majority, while those with 3, or 4 individuals are approximately 3.5 and 10.5 times less common, respectively. Larger networks, with a maximum number of 10, are at least 50 fold less common than those with 2 individuals. Table A1 summarizes the number of chains in each of the sex, age, and symptom classes observed in the Kanagawa and Hokkaido viral transmission networks. Overall, the chain distributions differed significantly between the two prefectures except for the chains for sex. The table demonstrated that age ≥60 homophily chains in the (3,3) class were more prevalent in Hokkaido than in Kanagawa (33% vs. 18%) while aged 30–59 homophily chains (2,2) were more common in Kanagawa than in Hokkaido 24% vs. 9%). Similarly, the proportion of asymptomatic homophily chains (1,1) was significantly higher in Kanagawa than in Hokkaido (49% vs. 2%), which is attributable to the fact that asymptomatic patients were less likely to transmit the virus than symptomatic patients in Kanagawa. Among the symptom homophily chains, Kanagawa demonstrated a higher proportion of the chains for most symptoms, including fever (58% vs. 29%), headache (3% vs. 1%), gastrointestinal (1.4% vs. 0.5%), mild/upper respiratory class (23% vs. 9%), severe/lower respiratory class (33% vs. 9%), and sensory disruption (3% vs. 2%).

**Table A1:** Distributions of Homophily * and Heterogeneous ** Chains in Viral Transmission Networks by Prefecture.

Variable	Class	Hokkaido		Kanagawa		*p*-Value
		(Total No. of Chains = 763)		(Total No. of Chains = 659)		
		No. of Chains	%	No. of Chains	%	
Sex						
Homophily Class	Female: (0, 0)	185	24.25	165	25.04	
	Male: (1, 1)	194	25.43	152	23.07	0.59
Heterogeneous class	Other: (1, 0) or (0, 1)	384	50.33	342	51.90	
Age						
Homophily Class	Age < 30: (1, 1)	26	3.41	89	13.51	
	30 ≤ Age < 60: (2, 2)	72	9.44	161	24.43	
	Age ≥ 60 (3, 3)	248	32.50	116	17.60	<0.001
Heterogeneous class	Other: (1, 2), (2, 1), (1, 3), (3, 1), (2, 3) or (3, 2)	417	54.65	293	44.46	
Asymptomatic Status						
Homophily Class	No: (0, 0)	277	36.30	563	85.43	
	Yes: (1, 1)	372	48.75	16	2.43	<0.001
Heterogeneous class	Other: (1, 0) or (0, 1)	114	14.94	80	12.14	
Fever						
Homophily Class	No: (0, 0)	433	56.75	63	9.56	
	Yes: (1, 1)	225	29.49	385	58.42	<0.001
Heterogeneous class	Other: (1, 0) or (0, 1)	105	13.76	211	32.02	
Headache						
Homophily Class	No: (0, 0)	683	89.52	491	74.51	
	Yes: (1, 1)	4	0.52	19	2.88	<0.001
Heterogeneous class	Other: (1, 0) or (0, 1)	76	9.96	149	22.61	
Body ache						
Homophily Class	No: (0, 0)	697	91.35	565	85.74	
	Yes: (1, 1)	9	1.18	7	1.06	<0.001
Heterogeneous class	Other: (1, 0) or (0, 1)	57	7.47	87	13.20	
Gastrointestinal						
Homophily Class	No: (0, 0)	716	93.84	485	73.60	
	Yes: (1, 1)	4	0.52	9	1.37	<0.001
Heterogeneous class	Other: (1, 0) or (0, 1)	43	5.64	165	25.04	
Mild/upper respiratory						
Homophily Class	No: (0, 0)	545	71.43	220	33.38	
	Yes: (1, 1)	70	9.17	151	22.91	<0.001
Heterogeneous class	Other: (1, 0) or (0, 1)	148	19.40	288	43.70	
Severe/lower respiratory						
Homophily Class	No: (0, 0)	543	71.17	265	40.21	
	Yes: (1, 1)	67	8.78	88	13.35	<0.001
Heterogeneous class	Other: (1, 0) or (0, 1)	153	20.05	306	46.43	
Sensory disruption						
Homophily Class	No: (0, 0)	678	88.86	485	73.60	
	Yes: (1, 1)	14	1.83	23	3.49	<0.001
Heterogeneous class	Other: (1, 0) or (0, 1)	71	9.31	151	22.91	

* Homophily chain refers to the situation where an infector and his/her infectee shares the same characteristics. ** Heterogeneous chain refers to the situation where an infector and his/her infectee have different characteristics.The chain number for each of the homophily classes is shown for Hokkaido and Kanagawa. The symptom homophily classes assessed were fever, headache, body ache, gastrointestinal issues (nausea and vomiting), upper respiratory involvement (cough, sneezing, rhinitis), lower respiratory involvement (dyspnea), and sensory disruption (anosmia and ageusia). We also assessed the number of homophily chains for age, sex, and asymptomatic status.
